# Twenty years of university-graduated osteopaths in Belgium: A cross-sectional survey

**DOI:** 10.1371/journal.pone.0359658

**Published:** 2026-10-05

**Authors:** David Zarka, Christian Denaeyer, Ludivine Lahaeye, Walid Salem, Ana Bengoetxea

**Affiliations:** 1 Osteopathic Sciences Research Unit (URSO), Faculty of Human Movement Sciences, Université libre de Bruxelles (ULB), Brussels, Belgium; 2 Département des Sciences de la Motricité, Haute Ecole Bruxelles – Brabant, Bruxelles, Belgium; Endeavour College of Natural Health, AUSTRALIA

## Abstract

**Background:**

In 2004, the Université libre de Bruxelles (ULB, Belgium) launched Europe’s first comprehensive university‑based osteopathy program, a six‑year curriculum intended to set an international standard for training osteopaths in the primary care management of functional musculoskeletal pain and disorders. This study describes the socio‑professional profile of ULB‑trained osteopaths in Belgium by graduation cohort.

**Methods:**

All contactable ULB graduates (n = 277; 83.9%) were invited to complete a voluntary, online cross‑sectional survey (49 items) disseminated via cohort‑personalized institutional emails between February and April 2024. Descriptive statistics were produced, and associations with time since graduation were examined.

**Results:**

A total of 156 graduates responded (56.3%), of whom 148 (94.9%) reported currently practicing as osteopaths. Respondents were predominantly female (60.9%) and aged 25–34 years (77.5%). Most practiced in French‑speaking Belgium (75.5%), while 17.6% worked abroad. The majority were full‑time self‑employed (92.6%), working in private offices (66.7%) and in multidisciplinary settings (68.6%), including private (53.2%) and public (15.4%) institutions. With increasing time since graduation, the number of concurrent workplaces decreased (p < 0.01), whereas financial independence (p < 0.01) and perceived professional achievement (p < 0.001) increased. Most osteopaths reported meeting their objectives within five years of practice.

**Conclusion:**

This first socio‑professional profile of university‑trained osteopaths in Belgium details where graduates practice and how career trajectories evolve across graduation cohorts. While the ULB program fosters integration of osteopaths in multidisciplinary teams, the absence of equivalent university education in the Dutch-speaking regions leads to important regional disparities. These findings may inform stakeholders and policy makers considering future regulation of osteopathic practice in Belgium and other countries.

## Introduction

Defined in 1874 in the United States (US), osteopathy is now well recognized as a person-centered, hands-on medical discipline based on biomedical screening, physical examination, and manual treatment, with a focus on holistic patient care [1]. Osteopathy can be beneficial for a wide range of conditions, with a primary focus on alleviating musculoskeletal pain and disorders [2–4]. It is widely practiced around the world and has been regulated in at least 13 Western countries [5]. In 2010, the World Health Organization established benchmarks for osteopathic clinical training [6], and in 2015, the European Committee for Standardisation defined standards to favor high-quality clinical practice, education, safety and ethics for 33 European countries [7]. However, significant variations persist in osteopathic practice worldwide. These differences stem in part from country-specific historical evolution, different educational pathways and clinical training approaches, as well as diverse legal frameworks and professional statuses [5].

In this context, two distinct yet related osteopathic professions with different scopes of practice are recognized. In the US, osteopathic physicians (D.O.) are fully trained medical doctors with unrestricted medical practice and prescribing rights. This means that manual practice is one of their medical tools among many, and that they can specialize in any branch of healthcare. Osteopathic physicians constitute a well-established and widely recognized professional group that is fully integrated into the healthcare system. Their numbers have increased by 70% over the past decade, and now account for more than 11% of all U.S. physicians (approximatively 150,000 practioners), with 43% practicing in specialized care [8]. In contrast, osteopaths outside the U.S. are trained specifically in frontline, primary osteopathic care, with a strong emphasis on hands-on patient care. As such, osteopaths are expected to refer patients appropriately when a condition requires therapeutic intervention that falls outside the scope of manual osteopathic practice [9]. Consequently, osteopathy outside the US remains an emerging profession characterized by a limited scope of practice and a legal framework that varies considerably across countries. A systematic review conducted by Ellwood and Carnes [10] reports on 14 surveys carried out to characterize the profile and clinical practices of osteopaths in different Western countries, including Australia, Benelux, Italy, Spain, Switzerland, Canada, Germany and the UK. Surveys on osteopaths’ profiles have also been conducted more recently in Portugal [11], Belgium [12], Austria [13] and France [14].

Despite variable osteopathic profiles and legal frameworks across countries outside the US, osteopathy has been identified as one of the most frequently consulted complementary healthcare practices in Western countries [10,15]. In Belgium for example, the annual patient count tripled between 2001 and 2024 reaching 12.4% of the population [16,17] with a satisfaction rate close to 90% [18]. Given its widespread use, ensuring the safety and quality of osteopathic care constitutes significant public health concern. In particular, the regulation of osteopathy as a form of primary care health profession raises questions about potential risks associated with misdiagnosis and certain manipulative procedures [18]. While spinal manipulation is considered safe when performed by appropriately trained professionals, adverse events may partly result from failures to identify contraindications or clinical red flags. Although these adverse events are rare [19], some studies suggest that a substantial proportion of them could be preventable through thorough and appropriate clinical screening [20]. Furthermore, indirect risks, such as delayed or missed diagnosis, may arise when clinical assessment is inadequate or when timely patient referral is not ensured. As such, several global reports emphasized the need for high-quality training programs and rigorous regulation of osteopathy profession [6,7].

To address these challenges, the first comprehensive university program in osteopathy in Europe was launched in 2004 at the *Université libre de Bruxelles* (ULB, Belgium). Designed for students with no prior healthcare training (Type I training), this six-year full-time program includes coursework shared with the medical curriculum and was developed jointly by the Faculty of Human Movement Sciences and the Faculty of Medicine to meet the highest standards expected of a primary, high-responsibility healthcare profession. It is structured as a bachelor’s degree (BSc, 3 years), a master’s degree (MSc, 2 years), and an additional specialized master’s degree in osteopathy (MSc.O, 1 year). It encompasses a comprehensive array of university courses in anatomy, biomechanics, physiology, pathology, semiology, psychology, as well as history and sociology of health (Fig 1). It involves extensive manual osteopathic practice training, internships in both private and hospital settings, and interprofessional collaborative practices.

**Fig 1:**
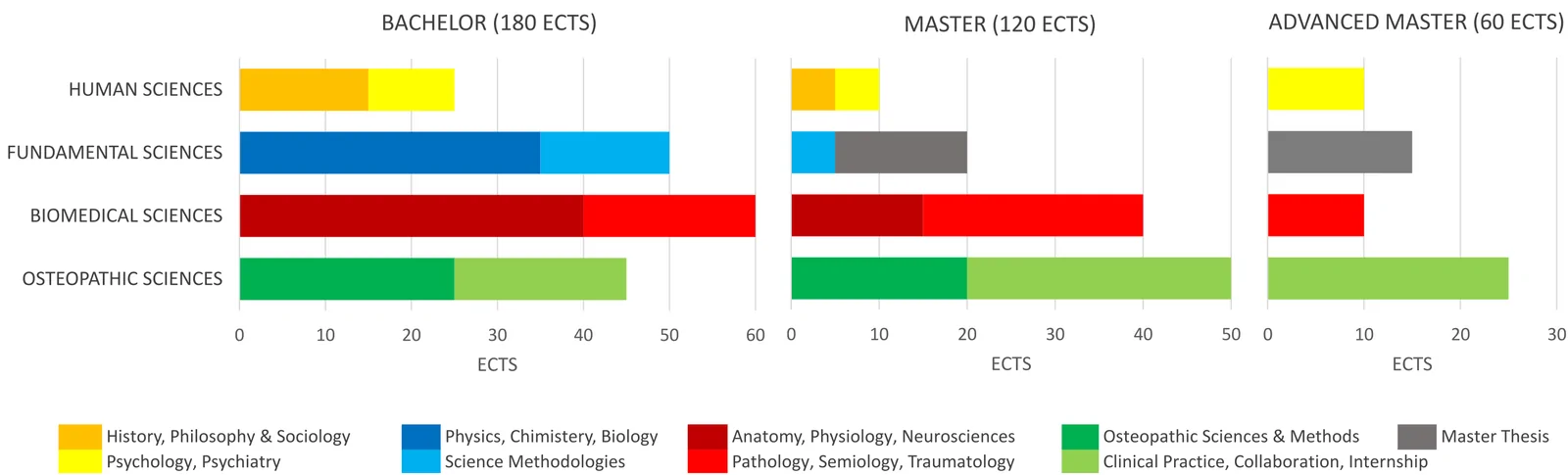
Description of the university program in osteopathy at the *Université libre de Bruxelles* (ULB, Belgium). ECTS: European Credit Transfer and Accumulation System. Credit in the ECTS system generally corresponds to 30 hours of work (lectures, tutorials, personal study, etc.), and 60 credits represent one full year of study (Bachelor’s or Master’s).

This advanced university program was developed to establish an international standard for training osteopaths in the primary management of functional musculoskeletal pain and disorders. Although five private part-time osteopathic education institutes (OEI) still operate in Belgium, the introduction of this full-time university program has significantly reshaped the landscape of Belgian osteopathy [12]. Between 2010 and 2023, 330 university-trained osteopaths have been educated to collaborate closely with other healthcare professionals and to provide primary care for functional musculoskeletal disorders. Twenty years after the launch of the program at ULB [21], a comprehensive survey was conducted to document the characteristics and clinical activities of university-graduated osteopaths (MSc.O) in Belgium. The present study constitutes the first component of this broader effort and focuses specifically on graduates’ sociodemographic profiles and professional status considering the year of their graduation. While osteopathy has not yet achieved legal recognition within the Belgian healthcare system, it is hypothesized that university-trained osteopaths have become increasingly integrated and recognized into conventional healthcare structures over time. Accordingly, this study examines variations in the geographical distribution, practice settings, and socioeconomic status of graduates across different cohorts. Ultimately, it aims to provide empirical data to better understand the role of university education in the integration and professional recognition of osteopaths in Belgium.

## Materials and methods

### Study design

To provide a comprehensive profile of university-graduated osteopaths, we conducted an online 2-part cross-sectional survey. The survey was distributed between the 1^st^ February and the 30^th^ April 2024. Designed using Google Forms platform, the survey included 85 items across 8 sections, combining multiple-choice and open-ended questions, and was administered as two separate single-measure questionnaires. The first questionnaire was designed to provide a comprehensive overview of the socio-professional characteristics of ULB osteopathy graduates since the first degrees were awarded in 2010. The second questionnaire was aimed at characterizing the clinical practices of these graduates. Both survey questionnaires were designed to be independent of each other and to ensure the complete anonymity of respondents. The present study is predicated on the first questionnaire (see *S1**_Files*) and focuses on the description of socio-professional situation of osteopaths trained at ULB according to their graduation date. However, two questions included in this study were imported from the second questionnaire (number of patients per week, and consultation time), since it also touched on the professional situation of practitioners. The study adheres to the reporting guidelines for research surveys as outlined by Wilson et al. [22].

### Questionnaire validation

The 2-part survey was developed by two investigators drawing on previously published instruments in osteopathy research [23,24], including elements adapted from the OPERA framework [12]. Item selection and questionnaire structure were guided by the primary objective of the study, namely to characterize the sociodemographic and socio-professional profiles of university-trained osteopaths, as well as their career trajectories. To ensure content relevance, domains commonly explored in prior osteopathic workforce surveys (e.g., demographics, professional status, practice characteristics, and career development) were retained and adapted to the specific context of a university-based training program. The first questionnaire comprised 49 items, divided into four thematic sections. Forty questions were mandatory, including 28 close-ended items with predefined response options (among which three used 10-point Likert scales) and 10 single-item open questions (word or numerical responses). Two additional mandatory single-item questions were imported from the second questionnaire for analytical purposes. Seven extended open-ended questions (two mandatory) were not included in the quantitative analysis but were used to collect complementary qualitative qualitative information, particularly regarding career changes.

The questionnaire was administered in French. As all graduates are proficient in this language given that the ULB osteopathic program is delivered in French, no additional (e.g., Dutch or German) version was developed. To enhance content validity and overall clarity, the whole survey (first and second parts) was subjected to a structured pre-testing process. First, three experts involved in osteopathic training reviewed the questionnaire to assess its relevance, completeness, and appropriateness in relation to the study objectives. Revisions were made to improve item wording and content coverage based on their feedback. Subsequently, four graduates from different cohorts (graduation years: 2010, 2015, 2019, 2023) were asked to test the questionnaire and provide additional feedback on clarity, terminology, and response formats. Each was asked to evaluate their understanding of the questions, wording, and technical terms. The instrument was iteratively refined until all items were consistently understood without difficulty. As the questionnaire was designed for descriptive and exploratory purposes, no formal psychometric validation (e.g., reliability or factor analysis) was conducted.

Participants were limited to one submission. The first page of the online platform provided standardized information on the study objectives, completion instructions, estimated duration, and ethical considerations. Respondents then had the possibility to fill out the questionnaires at their convenience and modify their answers using a back button. For questions related to their clinical practice, respondents were asked to refer to objective records (e.g., appointment schedules, financial summaries) over the past year where possible, or otherwise, provide their most accurate estimates. Duplicate responses were screened and excluded through cross-checking key variables (graduation years, gender, age, nationality, and postal code of the main place of practice) prior to analysis.

### Participants and recruitment

The osteopathy program at the *Université libre de Bruxelles* (ULB, Belgium) was established in 2004, with the first cohort completing their six-year training in 2010. Between 2010 and 2023, a total of 330 students graduated. All graduates were invited to participate on a voluntary basis by the secretariat of the *Osteopathic Sciences Research Unit* (URSO, Belgium) via cohort-personalized email invitation, using their public professional and/or student addresses. Out of the 330 alumni, 277 (83.9%) were successfully reached, while 53 (16.1%) could not be contacted directly due to unavailable contact details (Fig 2). To optimize the response rate, we followed recommendations from Shiyab et al. [25]. Two reminders were sent at two-week intervals to achieve the optimum response rate. Each graduate class also received a targeted email indicating the contacts we were missing from their cohort, with an invitation to forward the survey to these individuals through their own alumni networks. No form of financial compensation was provided. Even though responses were anonymous, participation may be regarded as quasi-voluntary, as recipients may have felt obliged to respond on account of their affiliation with the distributing institution. The study excluded osteopathic students. No professional associations or external organizations were involved in or sponsored the survey.

**Fig 2:**
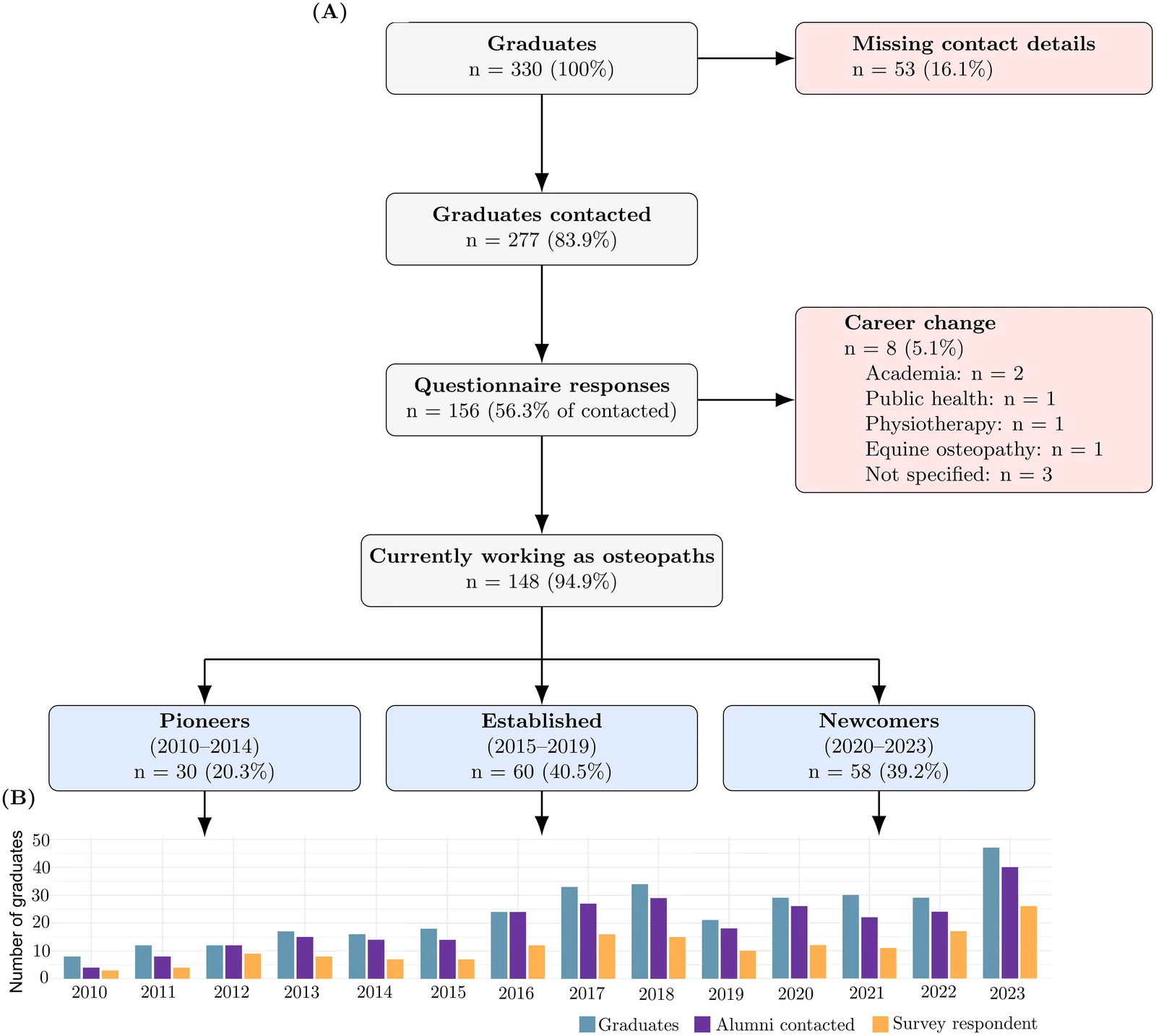
(A) Flowchart of the survey process. (B) Distribution of graduates, as well as contacted individuals, and questionnaire respondents across graduation years.

### Ethical statement

The study was conducted according to the guidelines of the Declaration of Helsinki. Ethical review and approval were waived for this study under the Belgian law, as the survey focused exclusively on healthcare professionals’ practices without any reference to patients [26,27]. Details of the study procedures were provided to the participants in the invitation e-mail and the survey’s introductory webpage. Alumni who volunteered to participate provided informed consent through participation by confirming having read and understood the survey’s introductory webpage. The questionnaire respected the anonymity and privacy of data in accordance with the European directive 2002/58/CE and the General Data Protection Regulation (GDPR) 2016/679. IP addresses were not disclosed to the research team. After the data collection process, the whole online survey and all associated responses were exported, manually erased, and the files deleted from Google Forms, Sheets, and Drive. No attempt was made to identify respondents, and no sensitive data was collected, such as name, surname, date of birth, fiscal code, email address, residency and professional address, nor genetic, biometric, healthcare, ethnic, racial and sexual-orientation-related data.

### Statistical analysis

According to survey features and recruitment strategies, response rates typically range between 17.1% and 43.4%, with the lowest response rate (17.1%) observed in online surveys without incentive [28]. Response rates tend to be higher in specialized healthcare contexts, with an average participation rate of 45.8% (± 25.0%) in web-based surveys among targeted health professionals worldwide, and 38.4% (± 14.0%) in Belgium [29]. Accordingly, it was hypothesized that between 81 and 173 graduates would respond to the survey. Given uncertainty in both sample size and its distribution across cohorts, responses were categorized into two to four groups based on year of graduation. This approach was chosen to balance temporal granularity with adequate group size, thereby ensuring both comparability across groups and interpretability of the results. In the final analysis, respondents were grouped into three cohorts according to their year of graduation (2010–2014, 2015–2019, and 2020–2023), hereafter referred to as “pioneers”, “established”, and “newcomers”.

Descriptive statistics were used to summarize sample characteristics. Categorical variables are presented as frequencies and percentages. Categorical variables were compared across these groups using Pearson’s Chi-square test, or Fisher’s exact test when expected cell counts were <5. Association strength was quantified with Cramér’s V. For quantitative or ordinal variables, inter-group differences were assessed using the non-parametric Kruskal–Wallis’s test, with epsilon-squared (ε²) reported as an effect size. Intra-individual comparisons of consultation fees (initial versus current) were performed using the paired Wilcoxon signed-rank test. Post-hoc analyses by category were adjusted for multiple testing with the Benjamini–Hochberg procedure. All tests were two-tailed, and statistical significance was set at *p* < 0.05. All statistical analyses were performed using R Studio (version 4.2.3).

## Results

The first questionnaire was completed by 156 graduates (56.3%). Of the respondents, 148 (94.9%) reported currently working as osteopaths, while 8 (5.1%) had transitioned to other careers (Fig 2). The mean response rate by graduation year was 56.7% [IC95: 50.8%; 62.5%]. Among the 148 practicing osteopathy, respondents were classified into three groups: *pioneers* (2010–2014, n = 30, 20.3%*)*, *established* (2015–2019, n = 60, 40.5%), and *newcomers* (2020–2023, n = 58, 39.2%). Accordingly, the mean response rate by groups was 58.5% [45.2%; 71.8%] for pioneers, 53.6% [44.3%; 62.8%] for established and 58.9% [49.8%; 68.0%] for newcomers (Fig 2). This stratification was used for comparative analyses in the subsequent questions studied. Detailed results are provided in Tables 1–3.

**Table 1:** Sociodemographic characteristics by group.

Categories	Total	Goups			V	*p*
		Pioneers	Established	Newcomers		
Sex % (n)					0.08	n.s.
Female	60.9 (95)	53.3 (16)	63.3 (38)	64.1 (41)	0.08	n.s.
Male	39.1 (61)	46.7 (14)	36.7 (22)	35.9 (23)	0.08	n.s.
Age, % (n)					0.70	***
20–24	1.3 (2)	0.0 (0)	0.0 (0)	3.0 (2)	0.13	n.s.
25–29	36.5 (57)	0.0 (0)	13.3 (8)	74.2 (49)	0.67	***
30–34	41.0 (64)	13.3 (4)	75.0 (45)	22.7 (15)	0.54	***
≥ 35	21.2 (33)	86.7 (26)	11.7 (7)	0.0 (0)	0.79	***
Nationality, % (n)					0.15	n.s.
Belgium	55.8 (86)	70.0 (21)	46.7 (28)	57.8 (37)	0.17	n.s.
France	37.0 (57)	30.0 (9)	46.7 (28)	31.2 (20)	0.17	n.s.
Other	7.2 (11)	0.0 (0)	6.7 (4)	10.9 (7)	0.11	n.s.
Country of practice, % (n)					0.11	n.s.
Belgium	79.5 (124)	80.0 (24)	73.3 (44)	84.9 (56)	0.11	n.s.
Brussels-Capital	51.4 (76)	39.3 (11)	49.1 (26)	55.6 (40)	0.22	n.s.
Flemish region	4.7 (7)	4.6 (1)	1.9 (1)	6.9 (5)	0.14	n.s.
Walloon region	43.9 (65)	56.1 (16)	49.0 (26)	37.5 (27)	0.19	n.s.
Abroad	17.6 (26)	20.0 (6)	26.7 (16)	15.1 (10)	0.11	n.s.
Professional status, % (n)					0.18	n.s.
Employee	1.4 (2)	3.6 (1)	0.0 (0)	1.6 (1)	0.11	n.s.
Self-employed (secondary occ.)	5.4 (8)	7.1 (2)	8.9 (5)	1.6 (1)	0.15	n.s.
Self-employed (main occ.)	87.2 (129)	75.0 (21)	89.3 (50)	90.6 (58)	0.17	n.s.
Company owner	6.1 (9)	14.3 (4)	1.8 (1)	6.3 (4)	0.18	n.s.
Type of practice setting**^+^**, % (n)					0.16	n.s.
Private office	66.7 (105)	86.7 (26)	71.7 (43)	60.9 (39)	0.14	n.s.
Private multidisciplinary center	53.2 (83)	36.7 (11)	50.0 (30)	78.1 (50)	0.34	**
Community health center	5.8 (9)	6.7 (2)	3.3 (2)	7.8 (5)	0.09	n.s.
Hospital	9.6 (15)	13.3 (4)	8.3 (5)	4.7 (3)	0.15	n.s.
Other	2.7 (4)	3.3 (1)	3.3 (2)	1.6 (1)	0.05	n.s.
Number of workplaces, % (n)					0.25	**
1	32.4 (48)	57.1 (16)	39.3 (22)	15.6 (10)	0.34	***
2	38.5 (57)	32.1 (9)	37.5 (21)	43.2 (27)	0.25	*
≥ 3	29.1 (43)	10.7 (3)	23.2 (13)	42.2 (27)	0.09	n.s.
Professional union member, % (n)	72.3 (107)	64.3 (18)	67.9 (38)	79.7 (51)	0.16	n.s.

Abbreviations: occ. = occupation; % = percentage; n = number of individuals; V = Cramér’s V; *p* = p-value; n.s. = not significant.

+ For this variable, percentages may exceed 100% due to multiple response options (i.e., multiple answers per individual).

Effect sizes are reported using Cramér’s V.; Significance levels: ^*^p < 0.05, ^**^p < 0.01, ^***^p < 0.001.

**Table 2:** Professional engagement and academic activities by group.

Categories	Total	Group			V	*p*
		Pioneers	Established	Newcomers		
Continuing education per year, % (n)					0.17	n.s.
0	10.8 (16)	7.1 (2)	8.9 (5)	12.5 (8)	0.08	n.s.
1	36.5 (54)	50.0 (14)	39.3 (22)	26.6 (17)	0.18	n.s.
2	30.4 (45)	25.0 (7)	23.2 (13)	39.1 (25)	0.18	n.s.
≥ 3	22.3 (33)	17.9 (5)	28.6 (16)	18.8 (12)	0.11	n.s.
Scientific literature, % (n)					0.14	n.s.
Never (or almost never)	21.6 (32)	21.4 (6)	25.0 (14)	18.8 (12)	0.06	n.s.
Rarely	40.5 (60)	28.6 (8)	39.3 (22)	46.9 (30)	0.14	n.s.
Regularly	26.4 (39)	32.1 (9)	23.2 (13)	26.6 (17)	0.07	n.s.
Often	10.1 (15)	14.3 (4)	12.5 (7)	6.2 (4)	0.11	n.s.
Always (or almost always)	1.4 (2)	3.6 (1)	0.0 (0)	1.6 (1)	0.11	n.s.
Teaching, % (n)					0.26	n.s.
College	0.7 (1)	3.6 (1)	0.0 (0)	0.0 (0)	0.29	n.s.
Private training institution	2.7 (4)	7.1 (2)	3.6 (2)	0.0 (0)	0.26	n.s.
University	13.5 (20)	25.0 (7)	14.3 (8)	7.8 (5)	0.17	n.s.
Research, % (n)					0.41	n.s.
Associated researcher	0.7 (1)	3.6 (1)	0.0 (0)	0.0 (0)	0.35	n.s.
Clinician-researcher	0.7 (1)	0.0 (0)	0.0 (0)	1.6 (1)	0.31	n.s.
Scientific collaborator	7.4 (11)	10.7 (3)	3.6 (2)	9.4 (6)	0.41	n.s.
PhD student	4.1 (6)	7.1 (2)	5.4 (3)	1.6 (1)	0.27	n.s.

Abbreviations: % = percentage; n = number of individuals; V = Cramér’s V; n.s. = not significant; *p* = p-value.

Effect sizes are reported using Cramér’s V. Significance levels: ^*^p < 0.05, ^**^p < 0.01, ^***^p < 0.001.

p-values are derived from Chi-square or Fisher’s exact tests.

**Table 3:** Practice and perception characteristics by group: consultation features, economic situation and fulfillment.

Categories	Total	Group			V	*p*
		Pioneers	Established	Newcomers		
Consultation time (min)**^+^**, % (n)					0.20	n.s.
*<* 45 min	18.8 (22)	40.9 (9)	15.9 (7)	11.8 (6)	0.28	*
45 min	48.7 (57)	40.9 (9)	52.3 (23)	49.0 (25)	0.08	n.s.
*>*45 min	32.5 (38)	18.2 (4)	31.8 (14)	39.2 (20)	0.16	n.s.
Price (€), mode [min, max]						
Initial	50 [30–70]	50 [45–50]	50 [40–60]	60 [50–70]	0.59	***
Current	60 [50–80]	60 [50–70]	60 [50–65]	60 [50–80]	0.03	n.s.
Patients per week**^+^**, median [IQR]						
Total patient	25 [20.0]	30 [16.2]	30 [17.8]	20 [12.5]	0.15	***
New patients	10 [9.0]	9 [8.0]	10 [9.0]	10 [8.0]	0.01	n.s.
Financial independance, % (n)					0.28	**
100%	50.9 (56)	81.3 (13)	58.1 (26)	34.0 (17)	0.34	**
70–90%	20.0 (22)	18.8 (3)	20.9 (9)	20.0 (10)	0.03	n.s.
40–60%	20.0 (22)	0.0 (0)	14.0 (6)	32.0 (16)	0.30	*
0–30%	9.1 (10)	0.0 (0)	4.7 (2)	14.0 (7)	0.20	n.s.
Secondary occupation, % (n)	27.0 (40)	42.9 (12)	23.2 (13)	21.0 (13)	0.19	n.s.
Patient goals achieved, % (n)					0.37	***
100%	43.2 (64)	62.1 (18)	51.7 (31)	22.7 (15)	0.34	***
70–90%	29.7 (44)	34.5 (10)	35.0 (21)	21.7 (13)	0.17	n.s.
40–60%	13.5 (20)	3.5 (1)	11.7 (7)	30.3 (20)	0.30	**
0–30%	13.5 (20)	0.0 (0)	1.7 (1)	27.3 (18)	0.35	***
Professional fulfillment, % (n)					0.15	n.s.
Not fulfilled at all	0.0 (0)	0.0 (0)	0.0 (0)	0.0 (0)	0.00	n.s.
Not very fulfilled	4.7 (7)	0.0 (0)	7.1 (4)	4.8 (3)	0.12	n.s.
Fairly fulfilled	51.4 (76)	39.2 (11)	48.2 (27)	60.3 (38)	0.16	n.s.
Very fulfilled	43.9 (65)	60.7 (17)	44.6 (25)	34.9 (22)	0.18	n.s.
Work-life balance, % (n)					0.16	n.s.
Not balanced at all	1.4 (2)	0.0 (0)	0.0 (0)	3.2 (2)	0.14	n.s.
Poorly balanced	10.8 (16)	14.3 (4)	5.4 (3)	12.7 (8)	0.12	n.s.
Fairly balanced	50.0 (74)	35.7 (10)	57.1 (32)	50.8 (32)	0.15	n.s.
Very balanced	37.8 (56)	50.0 (14)	42.9 (21)	33.3 (21)	0.12	n.s.

Abbreviations: % = percentage; n = number of individuals; € = euro; mode = most frequent observed value; V = Cramér’s V; *p* = p-value; n.s. = not significant. ^**+**^ For these variables, which come from the second questionnaire, the total sample size is 121: n = 22 for pioneers, n = 44 for established participants, and n = 55 for newcomers. Effect sizes: Cramér’s V for categorical variables. For continuous variables, effect size is reported as epsilon squared (*ε*^2^). p-values are derived from Chi-square or Fisher’s exact tests (categorical variables) and from Kruskal–Wallis tests (continuous variables). Significance levels: ^*^p < 0.05, ^**^p < 0.01, ^***^p < 0.001.

### Socio-demographic characteristics

Among respondents, 60.9% were female (H/F ratio: 0.64). The gender ratio was slightly higher in pioneers (0.88), although it did not differ between the three graduate groups (p = 0.20). Most respondents were aged between 25 and 34 years (77.5%). Belgian nationals represented 55.8% of the respondents, followed by French nationals at 37.0%. Other nationalities have also appeared over the graduation years, although no significant association with the groups has emerged (p = 0.09). Most graduates practiced in Belgium (79.5%, Table 1). Within Belgium, over half (51.4%) practiced in the Brussels-Capital Region, followed by the French-speaking Walloon region (43.9%). In contrast, only 4.7% of respondents practiced in the Dutch-speaking Flemish region. The density of osteopaths by province is illustrated in Fig. 3. Although not significant (p = 0.40), a higher proportion of younger graduates practicing in the Brussels-Capital Region (55.6% of *newcomers* vs. 39.3% of *pioneers*), whereas the opposite trend was observed in the Walloon Region (37.5% of *newcomers* vs. 56.1% of *pioneers*). The remaining respondents practicing abroad (17.6%) were well distributed across the groups. Detailed results are provided in Tables 1.

**Fig 3:**
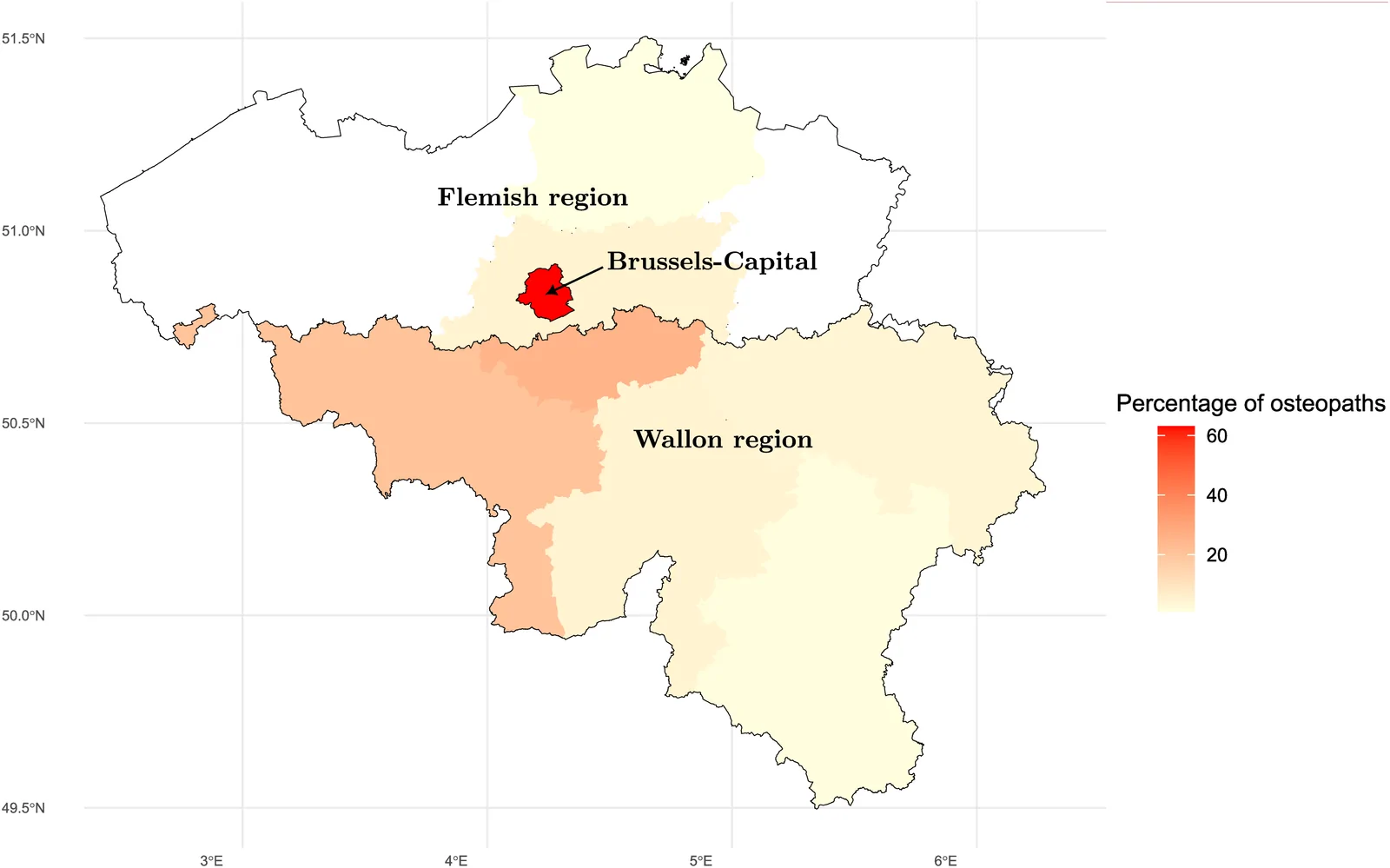
Geographic distribution of university-graduated osteopaths practicing by Belgian province.

### Work status and professionals’ activities.

The vast majority of respondents (92.6%) are self-employed osteopaths, with the majority (87.2%) doing so as their main occupation (Table 1). This proportion is slightly lower among pioneers (75.0%), in favor of private company ownership (14.3%). Two graduates reported being employed in a healthcare institution (one pioneer and one newcomer). Most graduates work in private offices (66.7%), but also in multidisciplinary centers (68.6%) including private (53.2%) and public (15.4%) institutions. No significant overall association was found between type of workplace and group (p = 0.33). Nevertheless, the proportion of graduates working in private multidisciplinary centers decreases significantly with the age of the ULB degree (V = 0.34, p < 0.01). The proportion of pioneers was indeed lower (36.7%) than that of the established (50.0%) and newcomers (78.1%) in the private multidisciplinary centers. Furthermore, the pioneers’ proportion in private offices (86.7%) and hospitals (13.3%) appeared to exceed that of the established (71.7% and 8.3% respectively) and newcomers (60.9% and 4.7% respectively). The number of workplaces also differed significantly across groups, according to the age of the ULB degree (V = 0.25, p < 0.01). Pioneers most often worked at a single location (57.1% vs. 15.6% among *newcomers*), whereas *newcomers* more frequently reported two or more workplaces (85.4% vs. 42.8% among *pioneers*). The majority of graduates (72.3%) were affiliated with a professional association, regardless of the group. Detailed results are provided in Table 1.

### Osteopathic teaching, research and lifelong learning.

Overall, no significant differences were found between the groups in terms of continuing education, scientific literature usage, teaching or research involvement (all p > 0.22, Table 2). The vast majority of respondents participated in continuing professional development courses, with at least one training course per year (89.2%). Most pioneers (50.0%) followed one course per year, while *established* (51.8%) and *newcomers* (57.9%) mostly followed two courses or more. A third (37.1%) of the graduates consult scientific literature regularly or more often. This proportion was marginally higher among the pioneers (50.0%) than among the established (35.7%) and newcomers (34.4%). Regarding teaching and research activities, 16.9% and 12.9% of graduates reported involvement in these activities respectively. The proportions of pioneers were also slightly higher in teaching (35.7%) and research (21.4%) than those of established (17.9% and 9.0% respectively) and newcomers (7.8% and 12.6% respectively), although the numbers in absolute terms remained relatively constant across groups. Detailed results are provided in Table 2.

### Consultation features, economic situation and fulfillment.

The most prevalent consultation duration among respondents is 45 minutes, irrespective of the group (48.7%, Table 3). However, a significant proportion of pioneers reported shorter consultations (40.9%, V = 0.28, p < 0.05) compared to established (15.9%) and newcomers (11.8%). In contrast, newcomers tend to offer longer consultations (39.2%) than established (31.8%) and pioneers (18.2%). While the pioneers and established started their activities with lower consultation fees than the newcomers (V = 0.59, p < 0.001), current fees were homogeneous across groups, typically around 60€ [50–80] per consultation. The median number of visits per week was 25 [20.0], of which 10 [9.0] were new patients. It differed significantly between groups (V = 0.15, p < 0.001), with newcomers reporting a lower total number of visits (20 [12.5]) than established (30 [16.2]) and pioneers (30 [17.2]).

Regarding the economic situation, most respondents (50.9%) reported achieving complete financial independence through their osteopathic practice alone (Table 3). However, a robust association was identified between financial independence and groups (V = 0.28, p < 0.01). A higher proportion of pioneers reported complete financial independence (81.3% vs 34.0% of newcomers), whereas newcomers reported more frequently moderate (60−40%) or lower (30−0%) level of financial independence (46.0% vs. 0.0% of *pioneers*). Among all graduates, 27.0% had a second occupation. Although not significant (V = 0.19, p = 0.06), a higher proportion of pioneers (42.9%) tend to have a second occupation compared to established (23.2%) and newcomers (21.0%).

In terms of professional goals and fulfillment, 43.2% of respondents reported having fully met their objectives in terms of patient numbers (Table 3). This proportion varied very significantly across groups (V = 0.37, p < 0.001). A higher proportion of pioneers reported achieving their target number of patients (62.1% vs. 22.7% of newcomers), whereas newcomers more frequently reported moderate (60–40%) or low (30–0%) levels of goal achievement (57.6% vs. 3.5% of pioneers). Most respondents described themselves as either fairly or very fulfilled professionally (95.3%) and fairly or very satisfied with their work-life balance (87.8%). These results did not differ significantly between groups, although slight increases appeared according to the age of the ULB degree in fulfilment and work-life balance.

## Discussion

### A university-graduated osteopaths’ profile

Our study enabled us to establish the sociodemographic and professional situation of university-graduated osteopaths in Belgium. The typical profile of a university-graduated osteopath is that of a French-speaking Belgian woman, aged between 30 and 34 years old, who is engaged in full-time self-employed osteopathic practice in both private office and multidisciplinary center in Brussels-Capital Region. She is a member of a professional union and is committed to ongoing professional development. According to the year of her graduation, she attained complete financial independence and achieved her patient goal after five years of practice. She reports having a good or very good work-life balance and feels fulfilled.

Interestingly, this profile contrast with the results of the Belgian OPERA study [12], which indicated that the typical osteopath in Belgium is a Dutch-speaking man, aged between 40 and 49, trained part-time after prior education in physiotherapy. This discrepancy can be explained by different target populations in our two studies. The 2018 OPERA survey targeted all Belgian osteopaths, irrespective of their training and academic degree. As such, most of the OPERA respondents had received prior professional qualification (70.3%, mainly as physiotherapist) and part-time (Type II) training in osteopathy (65.1%). In contrast, our study focusses on osteopaths who followed the full-time (Type I) university osteopathic program, with no prior healthcare training. The relatively recent implementation of this program inherently constrains the age distribution of respondents. Nevertheless, our three graduation groups correspond to three distinct age categories, showing that, unlike in Type II training, a limited number of former students were career changers or had already obtained a degree. In addition, only 4.7% of our respondents reported to work in the Dutch regions of Belgium, while the OPERA respondents were predominantly localized in these regions (52.5%). This uneven geographical distribution of university-graduated osteopaths is explained by the fact that ULB delivers its courses mainly in French and is the only university to offer an osteopathic training program in the country. There is no equivalent public osteopathic training in the Dutch part of the country. Therefore, the profile of university-graduated osteopaths differs from that of the average Belgian osteopath surveyed in 2018.

### Socio-demographics characteristics

Nevertheless, it is noteworthy that the data from van Dun et al. [12] showed a shift in both training type and gender ratio within the Belgian osteopaths’ population. While the number of woman osteopaths is increasing with a gender shift in the 20–29 and 30–39 age groups, full-time programs rose from 23.7% to 34.9% between 2013 and 2018 [12]. A clear gender difference was also observed in terms of the type of training, with 62.5% of women surveyed having completed a full-time training program, compared to 22.4% of men surveyed [12]. In this line, our results revealed an increase in the number of graduates and a shift in the gender ratio across cohorts. From a 1:1 ratio (one female for one male) among the pioneers, there was a transition to a 2:1 ratio among the established and newcomers. The female dominance in university-graduated osteopaths (60.1%) is consistent with the global trend towards a preponderance of women within the healthcare sector [30]. This female dominance in osteopaths is also observed in Australia (58.1%, [31]), Austria (70.7%) [13], Quebec (66.4%) [32], Germany (56.7%) [33], Switzerland (54.7%) [34], UK (59.1%) [35] and France (53.7%) [14]. A gender shift has also been observed in the 20–29 age group in Italy (33.3%) [23], Portugal (35.6%) [11] and Spain (40.1%) [36], although there is an overall male predominance in these countries.

While the ULB osteopathic program is the first at university level in Europe, another characteristic point is that 42.2% of responding graduates are of foreign nationality. The majority come from France (37.0%), where the osteopathy diploma is a professional qualification delivered exclusively by private institutions (not equivalent to a master’s degree) [14]. This ratio remains relatively stable across graduation groups, although the numbers of non-French foreign graduates tend to increase over the years. In contrast, only 17.6% of respondents reported working abroad, indicating that many expatriates stay in Belgium after their graduation. This trend can be explained by the lack of consistency between the legal frameworks of different countries. But it can also be explained by the six-year duration of the university program at an age when friendships and social situations can change significantly. Graduates may prefer to stay close to their social networks after graduation. In this regard, our data suggests a tendency for graduates to relocate from Brussels over time, with newcomers remaining in Brussels (55.6%) and pioneers settling in more distant French-speaking regions (56.1%). However, the present study did not specifically classify practice locations according to rural versus urban settings, which limits the ability to determine the proportion of osteopaths practicing in rural communities. Belgian administrative divisions encompass geographically and demographically heterogeneous areas, making such distinctions difficult without more refined spatial data. Future research adopting a more granular geographical approach would be valuable to characterize potential disparities in access to osteopathic care across settings. Such investigations would be particularly relevant considering evidence from general medical practice in Belgium, which highlights an imbalance in workforce distribution, with rural areas being relatively underserved [37,38].

### Work status, professional activities and career paths

In this regard, our data enables us to highlight the evolution over time of the socio-professional situation of graduates. Most graduates work as self-employed (87.2%) both in private offices and multidisciplinary centers (67.6%). However, the number of workplaces decreased significantly with seniority, alongside a significant increase in the number of patients per week, financial independence and achievement of professional goals. Thus, a greater proportion of pioneers, compared to other graduates, reported working in only one location (57.1%) in a private medical office (86.7%), owning their company (14.3%), achieved complete financial independence through their osteopathic practice alone (81.3%) and accomplished their professional objectives (62.1%). It is also interesting to note that a higher percentage of pioneers reported shorter consultation periods (40.9%), the presence of a secondary professional occupation (42.9%), a very satisfactory work-life balance (50.0%), and feeling very fulfilled (60.7%). These findings suggest that once expertise and financial security have been established, graduates tend to diversify their occupational activities while maintaining ongoing training in osteopathy. In this context, secondary professional occupations exhibited variability yet were predominantly related to health (e.g., acupuncture, yoga teacher, management of health center, etc.), or commitment in university (teaching and research assistant). Notably, a higher proportion of pioneers compared to other graduates reported regular or frequent engagement in reading scientific literature (50.0%) and participating in teaching (35.7%) and/or research (21.4%) activities. This phenomenon aligns with a broader trend that has been observed among younger generations, documented since 2010 [39], who have increasingly prioritized quality of life and meaningful occupation over the accumulation of substantial income.

In this line, it is noteworthy that the pioneers have reported an increase in their consultation fees over the years, from 50 [30–70] € to 60 [50–70] €. This increase remains below the average inflation rate in Belgium (2.65% per year) and is relatively comparable to the evolution of general medical consultation fees since 2011 [40]. Consequently, the current fee in osteopathy remains substantially lower than that of a general practitioner, which ranges between €30 and €33 [40] for an average consultation lasting approximately 15 minutes [41]. This fee trajectory appears to reflect an adjustment to inflationary pressures rather than a deliberate valorization of professional expertise or an explicit pursuit of financial gain. Such findings suggest that osteopaths may be inclined to maintain affordable fees for the population. Two primary factors may account for this tendency: first, the competitive environment within the profession, which likely incentivizes practitioners to keep prices accessible; and second, the absence of reimbursement by public social insurance, which reinforces the need to offer services at a level that remains financially attainable for patients.

In contrast to the pioneers, most established and newcomer graduates reported working in two or more workplaces (60.7% and 85.4%, respectively), with a substantial proportion affiliated with multidisciplinary centers (50.0% and 78.1%, respectively). Whereas osteopathy in Belgium has traditionally been practiced in solo private offices [12], the growing number of young graduates working within multidisciplinary teams may reflect a significant evolution in the field. This evolution could be explained by an increasing number of these centers actively recruiting osteopaths trained to academic standards. Notably, 9.6% of respondents reported working in hospital settings, and a subsequent investigation identified 28 osteopaths currently practicing across 18 hospital sites in Brussels [42]. In this context, the establishment of a university-level osteopathy program within the Faculty of Human Movement Sciences, in collaboration with the Faculty of Medicine at ULB, may have played a pivotal role in facilitating the integration of osteopathy into hospital environments and multidisciplinary teams. Notably, the university program provides interdisciplinary seminars that bring together osteopathy students and trainees from other health professions, that demonstrated impacts in collaboration skills among participants [43,44]. Other evidence from research on interprofessional education also highlights that a shared biomedical foundation improves communication and mutual understanding between different health professionals [45]. Furthermore, studies in osteopathy have shown that programs with a stronger biomedical orientation are perceived as more credible by medical doctors and other regulated health professionals, thereby creating greater opportunities for collaboration and institutional integration [46]. Taken together, these findings suggest that the ULB’s osteopathy program plays a crucial role in shaping the professional identity of osteopaths and in promoting their acceptance within multidisciplinary healthcare teams.

### Impact of the university program on the Belgian osteopathic landscape

Since its inception, the number of osteopathy graduates from ULB has grown steadily, increasing from 11 in 2011 to more than 40 in 2023, with an average annual growth of 18% per year. With a success rate of below 50%, this trend reflects both the growing attraction of science-informed osteopathy among young people and the rising proportion of graduated osteopaths without prior biomedical training. In line with this evolution, the proportion of osteopaths reporting full-time osteopathic training increased from 23.7% in 2013 [47] to 34.9% in 2018 [12]. Conversely, while 83% of respondents in 2013 [47] reported previous training in physical therapy, this figure declined to 65.8% in 2018 [12]. Although these data warrant updating, taken together they suggest that the establishment of a university-based osteopathy program has significantly reshaped the Belgian osteopathic landscape, fostering pathways of full-time osteopathic education without prior healthcare qualifications. This stands in contrast to countries such as Austria, Germany, Italy, Portugal, and Spain, where the majority of respondents reported part-time osteopathic training [11,13,23,33,36] and/or prior qualification as physical therapists [13,33,36].

As highlighted above, this evolution has also been characterized by a gender shift and a growing integration of osteopathy within multidisciplinary teams. To date, 330 university-trained osteopaths have been prepared to collaborate closely with other health professionals, providing primary care for functional musculoskeletal disorders and pain. Importantly, these graduates received training grounded in up-to-date scientific and biomedical foundation, shared with students from other biomedical disciplines, while critically revisiting traditional osteopathic concepts in light of contemporary scientific evidence [48]. Notably, among all European countries surveyed, Belgium stands out as the only one where the majority of respondents hold a master’s degree [12]. In terms of continuing education, the vast majority of respondents (89.2%) reported following at least a continuing education course per year, with more than two courses per year for both established (51.8%) and newcomers (57.9%). Furthermore, the observation that 4.1% of respondents are currently pursuing doctoral studies (excluding those who already hold a doctorate) suggests that university-based osteopathic education fosters a strong interest in research and promotes the development of a scientific approach to osteopathic practice. This also raises a major question regarding how the scientific-informed practice of university-trained osteopaths may differ from that of other osteopaths in Belgium.

### University-based osteopathic education as a benchmark for the profession

While D.O. in the US are fully integrated into the mainstream healthcare system as medical doctors, osteopathy has evolved into diverse professional models across Western countries. Although these models share some foundational principles which are now widely acknowledged within conventional healthcare (including a holistic, person-centered approach, the interrelationship between structure and function, and an emphasis on the body’s self-regulatory capacities), they differ significantly in scope of practice, training pathways, and levels of integration within healthcare systems. Consequently, the translation of these principles into contemporary clinical practice varies considerably across contexts. In many European settings, heterogeneity in training standards, ranging from part-time professional programs to more academically integrated curricula, has contributed to variability in professional identity, clinical reasoning, and integration within healthcare systems. In this context, the ULB program represents a distinctive model that seeks to move beyond these traditions. By embedding osteopathic training within a university framework shared with medical and health science curricula, it aligns osteopathic education with internationally recognized academic and biomedical standards, while updating core principles of osteopathic practice. The integration of foundational biomedical sciences, evidence-based practice, and interprofessional education supports the development of practitioners who are both grounded in up-to-date osteopathic principles and well-prepared to operate within modern healthcare systems.

From this perspective, the ULB program addresses several key challenges identified at the international level, including the need for standardized training, improved patient safety through robust clinical screening, the promotion of evidence-based and clinically reasoned practice, and enhanced integration into multidisciplinary care. While it does not aim to replicate the U.S. model of osteopathic medicine, it may reflect a convergent evolution toward a more academically grounded and healthcare-integrated form of osteopathy. Ultimately, the question of what it means to be an osteopath in Europe remains closely linked to the evolution of educational standards and regulatory frameworks. The present study suggests that university-based programs may play a pivotal role in shaping this identity, by promoting a model of practice that is scientifically informed, collaborative, and aligned with broader public health expectations

### Strengths and limitations

This study was the first to provide the specific profile of osteopaths who graduated from university, and examine their career paths, while this program aimed at setting an international standard for training osteopaths in primary care. Our findings highlighted the marked disparity in the distribution of university-graduated osteopaths across Belgium. Conversely, we showed positive progress in terms of integration into multidisciplinary healthcare settings and professional development of graduates. However, the study had also several limitations that should be acknowledged. While the response rate is substantial (56.3%), it is probable that participation is influenced by the alumni’s proximity to the university. Graduates who had a closer connection to the university or their former classmates probably responded in greater numbers than those who no longer had any connection with it. Therefore, the number of people who change careers after graduation may be underestimated. In contrast, the proportion of graduates actively engaged in research and teaching may be overestimated. This, however, is unlikely to have any significant impact on the analysis of changes according to the date of graduation. While drawing upon extant studies, the present survey was adapted to align more closely with the particularities of graduates following the osteopathic training program at ULB, which can limit comparability with previous surveys. As such, inquiries pertaining to professional identity are not incorporated, as the ULB training program was designed to cultivate osteopaths’ expertise exclusively, thereby fostering the development of their distinct professional identity and equipping them with the necessary competencies for professional recognition. Finally, as with any survey based on voluntary responses, the data may be influenced by respondent bias. Although respondents were asked to base their answers as much as possible on facts in cases of ambiguity, it is unclear to what extent this information was based on audited data.

## Conclusion

While the ULB training program in osteopathy is the first of its kind in Europe, the present study provides the first profile of osteopaths specifically trained in a university setting in Belgium. Since its inception in 2004, the ULB program has played a pivotal role in reshaping the Belgian osteopathic landscape. It has contributed to an increase in the number of professionals with exclusive osteopathic practices, as well as a gender shift towards greater female representation in the profession. As expected, the professional and economic situation of graduates has improved over the years. It is estimated that a period of five years is a requisite for achieving financial independence and attaining initial patient-related objectives. While promoting the development of a scientific approach to osteopathic practice, the ULB program also fosters integration of osteopaths in multidisciplinary teams, including in hospital settings. Nevertheless, the absence of equivalent university education in the Dutch-speaking regions leads to regional disparities, with a huge preponderance of university-graduated osteopaths practicing in Brussels and the French-speaking regions. The information provided could contribute to the body of data available to stakeholders and policymakers for the long-awaited regulation of the profession in Belgium.

## Supporting information

S1 File Survey first part. (PDF)
