# Supplementary material for: Twenty years of university-graduated osteopaths in Belgium: A cross-sectional survey
Source: PLoS One. 2026 Oct 5;21(10):e0359658. doi: 10.1371/journal.pone.0359658 (PMC13637917; doi:10.1371/journal.pone.0359658)
Supplement: Supplementary file 1 [file pone.0359658.s001.pdf]

SUPPLEMENTARY FILE

Twenty years of university-graduated osteopaths in Belgium : a cross-sectional survey

David Zarka[1,*], Christian Denaeyer[1], Ludivine Lahaeye[1],
Walid Salem[1,2] & Ana Bengoetxea[1]

[1] Osteopathic Sciences Research Unit (URSO), Faculty of Human Movement Sciences, Université Libre de Bruxelles (ULB), Brussels, Belgium

[2] Département des Sciences de la Motricité, Haute École Bruxelles–Brabant, Avenue Charles Schaller 91, 1160 Brussels, Belgium

*Corresponding author : David Zarka, `david.zarka@ulb.be`

Appendix : Questionnaire Used in the Study

1 Sociodemographic characteristics

1.1 Sex

❍ Female

❍ Male

1.2 Age

❍ 20-24

❍ 25-29

❍ 30-34

❍ 35-39

❍ 40-44

❍ 45-49

❍ 50-54

❍ 55-59

❍ 60-64

❍ 65-69

❍ 70+

1.3 Nationality

Short answer : ....

1.4 In which year did you obtain your Specialized Master's Degree in Osteopathy from ULB ?

Short answer : ....

1.5 Do you currently practice osteopathy professionally ?

❍ Yes (*Go to Section 3*)

❍ No (*Go to Section 2*)

2 You do not (or no longer) practice osteopathy professionally

2.1 How many years ago did you stop practicing osteopathy professionally ?

Short answer : ....

2.2 For how many years did you practice osteopathy professionally ?

Short answer : ....

2.3 In a few words (or more), could you describe your professional journey, major achievements, and current professional situation ?

Long answer : ....
....

2.4 In a few words (or more), what comments would you make regarding the strengths and/or limitations of your osteopathy training at ULB ?

Long answer : ....
....

3 You practice osteopathy professionally

3.1 In which country do you practice ?

❍ Belgium (*Go to Section 4*)

❍ France *(Go to Section 5*)

❍ Luxembourg *(Go to Section 5*)

❍ Switzerland *(Go to Section 5*)

❍ Other : ....*(Go to Section 5*)

4 You practice in Belgium

4.1 In which province(s) do you practice ?

❍ Antwerp

❍ Flemish Brabant

❍ Walloon Brabant

❍ Brussels-Capital Region

❍ West Flanders

❍ East Flanders

❍ Hainaut

❍ Liège

❍ Limbourg

❍ Luxembourg

❍ Namur

4.2 What is the postal code of your main practice location ? (Optional)

Short answer : ....

5 You practice outside Belgium

5.1 In which region do you practice ?

Short answer : ....

5.2 What is the postal code of your main practice location ? (Optional)

Short answer : ....

6 Professional status

6.1 Under which professional status do you practice ?

❍ Employee

❍ Self-employed (secondary occupation)

❍ Self-employed (primary occupation)

❍ Incorporated company

❍ Other : ....

6.2 In how many different practice settings do you work ?

❍ 1

❍ 2

❍ 3

❍ 4 or more

6.3 In which type(s) of practice setting(s) do you work ? (Multiple answers possible)

❍ Private practice

❍ Multidisciplinary clinic

❍ Community health center

❍ Hospital

❍ Other : ....

7 Professional association membership

7.1 Are you a member of a professional association ?

❍ Yes (*Go to Section 8*)

❍ No (*Go to Section 9*)

8 You are a member of a professional association ?

8.1 If you wish, please specify the professional association(s) of which you are a member. (Optional)

Short answer : ....

8.2 Have you held or do you currently hold a leadership position within a professional association (e.g., secretary, president, project manager) ?

❍ Yes

❍ No

8.3 If you wish, please describe any achievements or initiatives to which you contributed within your professional association. (Optional)

Short answer : ....

9 Continuing professional development

9.1 Do you regularly participate in continuing professional development (CPD) activities ?

❍ Yes

❍ No

9.2 How many CPD activities do you attend on average per year ?

❍ 0

❍ 1

❍ 2

❍ 3

❍ 4 or more

9.3 Do you participate in local peer-review or quality-improvement groups (e.g., GLEM, GLEMO) ?

❍ Yes

❍ No

9.4 How frequently do you consult the scientific literature ?

0	1	2	3	4	5
❍	❍	❍	❍	❍	❍
(Never or almost never)					(Always)

9.5 Have you participated in the organization of professional events (conferences, training courses, health promotion activities, etc.) ?

❍ Yes

❍ No

9.6 If you wish, please specify the professional events to which you contributed. (Optional)

Long answer : ....
....

10 Teaching activities

10.1 Do you teach in educational or professional training programs ?

❍ Yes (*Go to Section 11*)

❍ No (*Go to Section 12*)

11 Do you teach in educational or professional training programs ?

11.1 In which type(s) of institution(s) do you teach ? (Multiple answers possible)

❍ Private training organization

❍ University college

❍ University

❍ Other : ....

11.2 If you wish, please specify the title or subject area of your courses or training activities. (Optional)

Long answer : ....
....

12 Scientific research

12.1 Do you participate in scientific research activities ?

❍ Yes (*Go to Section 13*)

❍ No (*Go to Section 14*)

13 You take part in scientific research activities.

13.1 How do you contribute to scientific research ?

❍ Research collaborator

❍ Clinician

❍ PhD candidate

❍ Postdoctoral researcher

❍ Affiliate researcher

❍ Professor

❍ Other : ....

13.2 If you wish, please specify the title or topic of your research activities. (Optional)

Long answer : ....
....

14 Financial aspects

14.1 What was your consultation fee when you first started practicing ?

Short answer : ....

14.2 What is your current consultation fee ? If you use different fee schedules, please indicate your standard fee.

Short answer : ....

14.3 In addition to osteopathy, do you have another professional activity ?

❍ Yes (*Go to Section 15*)

❍ No (*Go to Section 16*)

15 Additional professional activity

15.1 If you wish, please specify your additional professional activity(ies). (Optional)

Short answer : ....

15.2 What proportion of your professional activity is represented by osteopathy compared with your additional professional activity ?

❍ 10% osteopathy / 90% additional professional activity

❍ 20% osteopathy / 80% additional professional activity

- ❍ 30% osteopathy / 70% additional professional activity
- ❍ 40% osteopathy / 60% additional professional activity
- ❍ 50% osteopathy / 50% additional professional activity
- ❍ 60% osteopathy / 40% additional professional activity
- ❍ 70% osteopathy / 30% additional professional activity
- ❍ 80% osteopathy / 20% additional professional activity
- ❍ 90% osteopathy / 10% additional professional activity

15.3 Regarding your osteopathic practice, have you achieved your target patient volume ?

- ❍ Yes
- ❍ No

15.4 If yes, how many years did it take you to achieve this objective ?

Short answer : ....

15.5 If no, what percentage of your target patient volume do you estimate having achieved ?

- ❍ 0%
- ❍ 10%
- ❍ 20%
- ❍ 30%
- ❍ 40%
- ❍ 50%
- ❍ 60%
- ❍ 70%
- ❍ 80%
- ❍ 90%

16 You do not have any additional professional activities

16.1 To what extent does your osteopathic practice provide sufficient financial independence to live without any additional source of income ?

❍ 0%

❍ 10%

❍ 20%

❍ 30%

❍ 40%

❍ 50%

❍ 60%

❍ 70%

❍ 80%

❍ 90%

❍ 100%

16.2 If you have achieved financial independence (90–100% in the previous question), how many years did it take you ?

Short answer : ....

16.3 Beyond financial independence, have you achieved your target patient volume ?

❍ Yes

❍ No

16.4 If yes, how many years did it take you to achieve your target patient volume ?

Short answer : ....

16.5 If no, what percentage of your target patient volume do you estimate having achieved ?

- ❍ 10%
- ❍ 20%
- ❍ 30%
- ❍ 40%
- ❍ 50%
- ❍ 60%
- ❍ 70%
- ❍ 80%
- ❍ 90%

17 Professional satisfaction

17.1 To what extent do you feel professionally fulfilled in your work as an osteopath ?

0	1	2	3	4	5	6	7	8	9	10
❍	❍	❍	❍	❍	❍	❍	❍	❍	❍	❍

0 = Completely dissatisfied and frustrated | 10 = By far the best profession in the world

17.2 To what extent do you consider your work–life balance to be satisfactory ?

0	1	2	3	4	5	6	7	8	9	10
❍	❍	❍	❍	❍	❍	❍	❍	❍	❍	❍

0 = Very poor | 10 = Excellent

17.3 Please provide any additional comments regarding your work–life balance, professional career, or osteopathy training.

Long answer : ....

....
