# Supplementary material for: Twenty years of university-graduated osteopaths in Belgium: A cross-sectional survey
Source: PLoS One. 2026 Oct 5;21(10):e0359658. doi: 10.1371/journal.pone.0359658 (PMC13637917; doi:10.1371/journal.pone.0359658)
Supplement: Supplementary file 2 [file pone.0359658.s002.docx]

Summary of the article: The article covers the profile of osteopaths based on year of graduation (pioneers vs established vs. newcomers). The demographic profile that showcased from this article is largely different than what was previously published, which is a novel finding given that osteopathic medicine is yet not adopted in Europe.

Major revision:

I believe in line 265 the author meant to state secondary occupation, otherwise the data does not match with what is in Table 3 (87.2% is listed for secondly occupation in the table.

Additional questions:

1- Given the variable practice across Belgium, how many osteopaths practice in rural communities. Is Flemish region rural?
2- If majority of respondents practice osteopathy as a secondary occupation, what is their main occupation? +
3- What is the similarity/difference between the osteopathic program in Europe Vs. the US, do they adopt the teants of osteopathic medicine? I think the discussion can improve by adding a relationship to osteopathic medicine as a whole (its inception and how/what does it mean to be an osteopath in Europe?
