# Supplementary material for: Twenty years of university-graduated osteopaths in Belgium: A cross-sectional survey
Source: PLoS One. 2026 Oct 5;21(10):e0359658. doi: 10.1371/journal.pone.0359658 (PMC13637917; doi:10.1371/journal.pone.0359658)
Supplement: Supplementary file 3 [file pone.0359658.s004.docx]

Dear Dr. Wilkinson,

Thank you for your evaluation of our manuscript entitled *“Twenty years of university-graduated osteopaths in Belgium: a cross-sectional survey”* (Manuscript ID: PONE-D-26-07875), and for the opportunity to revise and resubmit our work.

We have carefully considered all comments provided by the reviewers and have revised the manuscript accordingly. In particular, we have:

- Substantially revised the Introduction to clarify the rationale of the study and better align it with its descriptive objectives;
- Expanded the Methods section to provide a more detailed description of survey development, including item selection, structure, and pre-testing procedures;
- Refined the Discussion to improve interpretation of the findings and to better articulate their implications for workforce planning, regulation, and future research;
- Addressed all specific comments and corrected minor inconsistencies identified during review.

We believe that these revisions have significantly strengthened the clarity, coherence, and scientific rigor of the manuscript.

Please find attached:

- A detailed “Response to Reviewers” document addressing each comment point by point, with responses highlighted in red;
- A marked-up version of the manuscript highlighting all changes;
- A clean version of the revised manuscript.

We thank you and the reviewers for your constructive feedback and consideration of our work. We hope that the revised version meets the journal’s expectations and we remain at your disposal for any further clarification.

Kind regards,

David Zarka,

on behalf of all authors

**Review Comments to the Author**

Reviewer #1: General

Interesting study characterizing the graduates of a relatively new (and distinct) training program. The survey information is useful in that context; however, the intro does not set the stage particularly well and I think could use better justification for why this was done. As it stands, the intro feels like it spends most of its time arguing for standardization or training and explaining the unique (in Belgium) approach of the university in question. The paper itself does not really touch on this. Some attention in the discussion could also be given to what we can do with this data down the line. There could be better explanation of the survey development in the Methods (was there a pilot survey done, how were questions selected, rationale of grouping, etc). Otherwise, generally, well written. Results and conclusions are reasonable. Citations are appropriate.

- Authors’ response:

We thank the reviewer for the positive assessment of our study and for the constructive and insightful suggestions. In response, we have substantially revised and streamlined the Introduction to better justify the rationale of the study and to ensure closer alignment with its descriptive objectives. In particular, we have reduced the emphasis on standardisation and training models, and instead explicitly highlighted the underlying knowledge gap, namely the lack of empirical data on the socio-professional characteristics, integration, and career trajectories of university-trained osteopaths. We have also clarified and strengthened the Methods section, providing more detailed information on survey development, including the rationale for item selection, the use of previously published instruments, the structuring of the questionnaire, and the pre-testing process involving both experts and graduates. In addition, the Discussion has been expanded and refined to better articulate the interpretation of the findings and their implications, including how these data may inform workforce planning, professional regulation, and future research, as well as to more clearly distinguish structural factors from cohort-related observations. Overall, we believe that these revisions have improved the clarity, coherence, and relevance of the manuscript, and have ensured a better alignment between the study background, objectives, methods, and findings. Please find below our detailed point-by-point responses.

Major Comments

Lines 47-53: This is useful background information on the differences between US physician DOs and the non-physician osteopaths outside the US. But I don’t see a direct tie-in to your study, since your focus is the European/international model of osteopathy. The way it is written, I’m not sure if this is an aside or if you are making a commentary on the differences in training and scope. Perhaps some reframing to more directly compare training would make sense, especially since so little relative attention is given in this paragraph to the differences (other than saying that non-US osteopaths are “trained specifically in frontline, primary osteopathic care”) and why they might be relevant here.

- Authors’ response:

We thank the reviewer for this comment and agree that the original paragraph did not clearly articulate its relevance to the study. In the revised manuscript, we have reframed this section to more clearly position the comparison between U.S. osteopathic physicians and non-U.S. osteopaths as contextual background on differing training pathways and scopes of practice. We now clarify that this distinction serves to situate the European/international model of osteopathy, within which the ULB program operates, and to highlight how differences in training structure and clinical responsibilities may influence professional roles and integration into healthcare systems. In addition, the Discussion has been expanded through the introduction of a dedicated section (“University-based osteopathic education as a benchmark for the profession”), where these distinctions are further elaborated. This section situates the European model within the broader framework of osteopathic medicine, outlining both shared foundational principles and key differences with the U.S. model, and discussing how university-based training may contribute to a more integrated and academically grounded form of osteopathic practice in Europe. Together, these revisions strengthen the conceptual framing of the study and provide a clearer rationale for examining graduates’ socio-professional trajectories.

Lines 74-77: This is a somewhat vague and I think problematic claim. It is not clear how you are defining “non-exclusive osteopaths” and why you would assume that that would “impede the development of advanced diagnostic expertise” or “potentially increase(ing) the risk of accidents”. The cited study asked whether practitioners felt they were primarily osteopaths vs healthcare professionals but this is in the context of Australia’s legal environment where (as I understand it – at least at the time of the cited study) only physicians and physical therapists can perform osteopathic manipulations. What’s more in the US at least, osteopathic physicians have essentially always been physicians and are almost by definition “non-exclusive”. I have yet to see any data that shows worse training, expertise, or outcomes and that does not seem to be the goal of your paper. Please see below comment as well.

- Authors’ response:

We thank the reviewer for this important comment. The original wording was indeed **imprecise and potentially misleading**, particularly regarding the use of the term “non-exclusive osteopaths.”. This statement has been **removed**, and the paragraph has been **restructured** to avoid speculative interpretations. The revised version focuses instead on **patient safety, clinical screening, referral practices, and the diversity of training and regulatory frameworks**, providing a more accurate and evidence-based context aligned with the study objectives.

Lines 110-113: Your introduction delves quite deeply into the training of osteopaths in Belgium, how it compares to other areas, and generally seems to be making an argument for more rigorous training in a medical model. But your study aims seem to be more of a descriptive/exploratory survey of graduates. I would almost expect, based on the bulk of the intro, that you were examining outcomes, competency, continuing education, role in the larger healthcare system, etc. I do not get a good sense of why this is important or an open question. I think the intro needs to be reframed to answer this.

- Authors’ response:

We agree that the original Introduction placed disproportionate emphasis on training models, which could suggest an evaluative approach beyond the scope of the study. In the revised manuscript, the Introduction has been restructured and shortened, with a clearer focus on the descriptive aim of the study, namely to document the socio-professional characteristics, integration, and career trajectories of graduates. Background information on training has been refocused to support the research question, with greater emphasis on the lack of empirical data in this area, rather than on advocating for a particular educational model.

Minor Comments

Line 67: I would consider changing “unconventional” to “complementary”.

We agree with this suggestion and have replaced “unconventional” with “complementary” to align with current terminology and improve precision.

Line 80: What public health issue specifically?

We agree that the reference to “this public health issue” was unclear. In the revised manuscript, this has been clarified and replaced with a more explicit description, referring to patient safety, appropriate referral pathways, and the regulation of osteopathic practice. This revision improves clarity and ensures consistency with the concepts developed in the preceding sentences.

Line 83: When you say “equivalent to medical training”, do you mean in time or content? The implication seems to be both, but that is not clear from your breakdown of the curriculum. Is the intent here to imply that this model of training is overall equivalent to the training a medical doctor would receive (as it is in the US?).

We thank the reviewer for this clarification. The statement referring to “equivalent to medical training” has been removed and replaced with a more precise description of the program, clarifying its duration and academic structure without implying equivalence in scope of practice or professional status.

Line 137: This section talks about validation, but how was the survey developed? What rationale formed the basis for the questions?

This section has been substantially expanded to clarify how the survey was developed. The revised manuscript now details the use of previously published instruments, the rationale for item selection, and the thematic structuring of the questionnaire, aligned with the study objectives.

Lines 230 (Figure 2) and 236-239: What are these groups “Pioneers”, “Established”, “Newcomers”? Why were they grouped this way? This should be specified in the methods. It seems somewhat arbitrary.

We agree that the grouping required clearer justification. The definition and rationale for these groups have now been explicitly described in the Methods section (Statistical analysis subsection) as cohort-based groupings reflecting time since graduation. We also clarified that the number and composition of groups were determined a priori based on expected response distribution, and that this approach was chosen to balance temporal granularity with adequate group size, thereby ensuring both comparability across groups and interpretability of the results.

Lines 248-249: This seems somewhat obvious, people who went to school more recently tend to be younger.

This statement has been removed to improve conciseness and avoid redundancy.

Lines 369-370: Also worth mentioning that your university only started offering this program in 2004, which itself likely influences the age of respondents (especially since it is a primary/entry level degree).

We thank the reviewer for this relevant observation. We have added clarification in the Discussion to explicitly acknowledge that the relatively recent implementation of the ULB program inherently constrains the age distribution of respondents. At the same time, we have clarified that the cohort-based grouping still reflects meaningful differences, as only a limited proportion of graduates were career changers or had previously obtained another degree. This addition improves the interpretation of the results by distinguishing structural effects related to the training pathway from cohort-related characteristics.

Reviewer #2: Summary of the article: The article covers the profile of osteopaths based on year of graduation (pioneers vs established vs. newcomers). The demographic profile that showcased from this article is largely different than what was previously published, which is a novel finding given that osteopathic medicine is yet not adopted in Europe.

- Authors’ response:

We thank the reviewer for this summary and for emphasising the novelty of the findings. We agree that the cohort-based differences observed in this study reveal a socio-demographic and professional profile that contrasts with previously published surveys. In the revised manuscript, we have ensured that this contribution is more clearly positioned within the context of **heterogeneity in osteopathic training models across Europe,** rather than a lack of adoption of osteopathy per se. In particular, we emphasize the **distinctive nature of primary-entry, university-based training programs,** which remain relatively uncommon and may contribute to the differences observed in graduate profiles.

Major revision:

I believe in line 265 the author meant to state secondary occupation, otherwise the data does not match with what is in Table 3 (87.2% is listed for secondly occupation in the table.

- Authors’ response:

We thank the reviewer for carefully identifying this inconsistency. **The discrepancy resulted from an error in Table 3, where the percentage (87.2%) was incorrectly attributed to secondary occupation, while it actually corresponds to osteopathy as the primary occupation.** In the revised manuscript, we have corrected Table 3 accordingly and aligned the terminology throughout the Results and Discussion sections. We have also carefully cross-checked all related text and tables to ensure full consistency between reported values and their interpretation.

Additional questions:

1- Given the variable practice across Belgium, how many osteopaths practice in rural communities. Is Flemish region rural?

The present study did not specifically classify practice locations according to rural versus urban settings, which limits our ability to estimate the proportion of osteopaths practicing in rural communities. As clarified in the revised manuscript, our geographical analysis is based on administrative regions and provinces, which encompass heterogeneous urban and rural areas and therefore do not allow for a precise distinction at this level. We have added a statement in the Discussion acknowledging this limitation and highlighting the need for future studies using more granular geographical data to better assess potential disparities in access to osteopathic care across settings. This issue is particularly relevant in light of evidence from general medical practice in Belgium, where workforce distribution remains uneven across urban and rural areas.

2- If majority of respondents practice osteopathy as a secondary occupation, what is their main occupation?

The apparent confusion arose from an error in Table 3, which has now been corrected. The majority of respondents reported osteopathy as their primary occupation. Among those reporting a secondary occupation, activities were heterogeneous and predominantly health-related, including physiotherapy-related activities, sports instruction, yoga, acupuncture, or management of health centers, as well as academic roles such as teaching or research assistance.

3- What is the similarity/difference between the osteopathic program in Europe Vs. the US, do they adopt the teants of osteopathic medicine? I think the discussion can improve by adding a relationship to osteopathic medicine as a whole (its inception and how/what does it mean to be an osteopath in Europe?

We thank the reviewer for this insightful suggestion. We agree that this broader perspective strengthens the interpretation of our findings. In the revised manuscript, we have expanded the Discussion to more explicitly situate European osteopathy within the broader historical and conceptual framework of osteopathic medicine. We now clarify that European osteopathy and U.S. osteopathic medicine share common historical roots and foundational principles (such as a holistic, person-centered approach, the interrelationship between structure and function, and an emphasis on the body’s self-regulatory capacities) while differing substantially in training pathways, scope of practice, and regulatory status. We have also added a dedicated discussion on what it means to practice osteopathy in the European context, emphasizing its role as a primary-contact, non-physician profession, and highlighting how these characteristics have informed the development of the university-based program studied here.
