# Supplementary material for: Twenty years of university-graduated osteopaths in Belgium: A cross-sectional survey
Source: PLoS One. 2026 Oct 5;21(10):e0359658. doi: 10.1371/journal.pone.0359658 (PMC13637917; doi:10.1371/journal.pone.0359658)
Supplement: Peer review history

# Peer review: Decision Letter 0

**DOI:** 10.1371/journal.pone.0359658.r001

**Authors:** Jenny Wilkinson

15 Apr 2026

Dear Dr. Zarka,

Thank you for submitting your manuscript to PLOS ONE. After careful consideration, we feel that it has merit but does not fully meet PLOS ONE’s publication criteria as it currently stands. Therefore, we invite you to submit a revised version of the manuscript that addresses the points raised during the review process.

Please submit your revised manuscript by May 30 2026 11:59PM. If you will need more time than this to complete your revisions, please reply to this message or contact the journal office at plosone@plos.org. When you're ready to submit your revision, log on to https://www.editorialmanager.com/pone/ and select the 'Submissions Needing Revision' folder to locate your manuscript file.

- A letter that responds to each point raised by the academic editor and reviewer(s). You should upload this letter as a separate file labeled 'Response to Reviewers'.
- A marked-up copy of your manuscript that highlights changes made to the original version. You should upload this as a separate file labeled 'Revised Manuscript with Track Changes'.
- An unmarked version of your revised paper without tracked changes. You should upload this as a separate file labeled 'Manuscript'.

If you would like to make changes to your financial disclosure, please include your updated statement in your cover letter. Guidelines for resubmitting your figure files are available below the reviewer comments at the end of this letter.

If applicable, we recommend that you deposit your laboratory protocols in protocols.io to enhance the reproducibility of your results. Protocols.io assigns your protocol its own identifier (DOI) so that it can be cited independently in the future. For instructions see: https://journals.plos.org/plosone/s/submission-guidelines#loc-laboratory-protocols. Additionally, PLOS ONE offers an option for publishing peer-reviewed Lab Protocol articles, which describe protocols hosted on protocols.io. Read more information on sharing protocols at https://plos.org/protocols?utm_medium=editorial-email&utm_source=authorletters&utm_campaign=protocols.

As the corresponding author, your ORCID iD is verified in the submission system and will appear in the published article. PLOS supports the use of ORCID, and we encourage all coauthors to register for an ORCID iD and use it as well. Please encourage your coauthors to verify their ORCID iD within the submission system before final acceptance, as unverified ORCID iDs will not appear in the published article. *Only*  the individual author can complete the verification step; PLOS staff *cannot*  verify ORCID iDs on behalf of authors.

We look forward to receiving your revised manuscript.

Kind regards,

Jenny Wilkinson, PhD

Academic Editor

PLOS One

Journal requirements:

When submitting your revision, we need you to address these additional requirements.

1. Please ensure that your manuscript meets PLOS ONE's style requirements, including those for file naming. The PLOS ONE style templates can be found at

https://journals.plos.org/plosone/s/file?id=wjVg/PLOSOne_formatting_sample_main_body.pdf and

https://journals.plos.org/plosone/s/file?id=ba62/PLOSOne_formatting_sample_title_authors_affiliations.pdf

2. Your ethics statement should only appear in the Methods section of your manuscript. If your ethics statement is written in any section besides the Methods, please delete it from any other section.

If the reviewer comments include a recommendation to cite specific previously published works, please review and evaluate these publications to determine whether they are relevant and should be cited. There is no requirement to cite these works unless the editor has indicated otherwise.

[Note: HTML markup is below. Please do not edit.]

Reviewers' comments:

Reviewer's Responses to Questions

**Comments to the Author**

1. Is the manuscript technically sound, and do the data support the conclusions?

Reviewer #1: Yes

Reviewer #2: Yes

**********

2. Has the statistical analysis been performed appropriately and rigorously?

Reviewer #1: Yes

Reviewer #2: Yes

**********

3. Have the authors made all data underlying the findings in their manuscript fully available?

The PLOS Data policy

Reviewer #1: Yes

Reviewer #2: Yes

**********

4. Is the manuscript presented in an intelligible fashion and written in standard English?

Reviewer #1: Yes

Reviewer #2: Yes

**********

Reviewer #1: General

Interesting study characterizing the graduates of a relatively new (and distinct) training program. The survey information is useful in that context; however, the intro does not set the stage particularly well and I think could use better justification for why this was done. As it stands, the intro feels like it spends most of its time arguing for standardization or training and explaining the unique (in Belgium) approach of the university in question. The paper itself does not really touch on this. Some attention in the discussion could also be given to what we can do with this data down the line. There could be better explanation of the survey development in the Methods (was there a pilot survey done, how were questions selected, rationale of grouping, etc). Otherwise, generally, well written. Results and conclusions are reasonable. Citations are appropriate.

Major Comments

Lines 47-53: This is useful background information on the differences between US physician DOs and the non-physician osteopaths outside the US. But I don’t see a direct tie-in to your study, since your focus is the European/international model of osteopathy. The way it is written, I’m not sure if this is an aside or if you are making a commentary on the differences in training and scope. Perhaps some reframing to more directly compare training would make sense, especially since so little relative attention is given in this paragraph to the differences (other than saying that non-US osteopaths are “trained specifically in frontline, primary osteopathic care”) and why they might be relevant here.

Lines 74-77: This is a somewhat vague and I think problematic claim. It is not clear how you are defining “non-exclusive osteopaths” and why you would assume that that would “impede the development of advanced diagnostic expertise” or “potentially increase(ing) the risk of accidents”. The cited study asked whether practitioners felt they were primarily osteopaths vs healthcare professionals but this is in the context of Australia’s legal environment where (as I understand it – at least at the time of the cited study) only physicians and physical therapists can perform osteopathic manipulations. What’s more in the US at least, osteopathic physicians have essentially always been physicians and are almost by definition “non-exclusive”. I have yet to see any data that shows worse training, expertise, or outcomes and that does not seem to be the goal of your paper. Please see below comment as well.

Lines 110-113: Your introduction delves quite deeply into the training of osteopaths in Belgium, how it compares to other areas, and generally seems to be making an argument for more rigorous training in a medical model. But your study aims seem to be more of a descriptive/exploratory survey of graduates. I would almost expect, based on the bulk of the intro, that you were examining outcomes, competency, continuing education, role in the larger healthcare system, etc. I do not get a good sense of why this is important or an open question. I think the intro needs to be reframed to answer this.

Minor Comments

Line 67: I would consider changing “unconventional” to “complementary”.

Line 80: What public health issue specifically?

Line 83: When you say “equivalent to medical training”, do you mean in time or content? The implication seems to be both, but that is not clear from your breakdown of the curriculum. Is the intent here to imply that this model of training is overall equivalent to the training a medical doctor would receive (as it is in the US?).

Line 137: This section talks about validation, but how was the survey developed? What rationale formed the basis for the questions?

Lines 230 (Figure 2) and 236-239: What are these groups “Pioneers”, “Established”, “Newcomers”? Why were they grouped this way? This should be specified in the methods. It seems somewhat arbitrary.

Lines 248-249: This seems somewhat obvious, people who went to school more recently tend to be younger.

Lines 369-370: Also worth mentioning that your university only started offering this program in 2004, which itself likely influences the age of respondents (especially since it is a primary/entry level degree).

Reviewer #2: Summary of the article: The article covers the profile of osteopaths based on year of graduation (pioneers vs established vs. newcomers). The demographic profile that showcased from this article is largely different than what was previously published, which is a novel finding given that osteopathic medicine is yet not adopted in Europe.

Major revision:

I believe in line 265 the author meant to state secondary occupation, otherwise the data does not match with what is in Table 3 (87.2% is listed for secondly occupation in the table.

Additional questions:

1- Given the variable practice across Belgium, how many osteopaths practice in rural communities. Is Flemish region rural?

2- If majority of respondents practice osteopathy as a secondary occupation, what is their main occupation? +

3- What is the similarity/difference between the osteopathic program in Europe Vs. the US, do they adopt the teants of osteopathic medicine? I think the discussion can improve by adding a relationship to osteopathic medicine as a whole (its inception and how/what does it mean to be an osteopath in Europe?

**********

what does this mean?). If published, this will include your full peer review and any attached files.

If you choose “no”, your identity will remain anonymous but your review may still be made public.

**Do you want your identity to be public for this peer review?**  For information about this choice, including consent withdrawal, please see our Privacy Policy

Reviewer #1: **Yes:**  Alfred Amendolara

Reviewer #2: No

**********

[NOTE: If reviewer comments were submitted as an attachment file, they will be attached to this email and accessible via the submission site. Please log into your account, locate the manuscript record, and check for the action link "View Attachments". If this link does not appear, there are no attachment files.]

To ensure your figures meet our technical requirements, please review our figure guidelines: https://journals.plos.org/plosone/s/figures

You may also use PLOS’s free figure tool, NAAS, to help you prepare publication quality figures: https://journals.plos.org/plosone/s/figures#loc-tools-for-figure-preparation.

NAAS will assess whether your figures meet our technical requirements by comparing each figure against our figure specifications.

Attachment Submitted filename: Review of ostopathy program in Belgium.docx

# Author comment: Author response to Decision Letter 1

**DOI:** 10.1371/journal.pone.0359658.r002

19 Jun 2026

Dear Dr. Wilkinson,

Thank you for your evaluation of our manuscript entitled “Twenty years of university-graduated osteopaths in Belgium: a cross-sectional survey” (Manuscript ID: PONE-D-26-07875), and for the opportunity to revise and resubmit our work.

We have carefully considered all comments provided by the reviewers and have revised the manuscript accordingly. In particular, we have:

• Substantially revised the Introduction to clarify the rationale of the study and better align it with its descriptive objectives;

• Expanded the Methods section to provide a more detailed description of survey development, including item selection, structure, and pre-testing procedures;

• Refined the Discussion to improve interpretation of the findings and to better articulate their implications for workforce planning, regulation, and future research;

• Addressed all specific comments and corrected minor inconsistencies identified during review.

We believe that these revisions have significantly strengthened the clarity, coherence, and scientific rigor of the manuscript.

Please find attached:

• A detailed “Response to Reviewers” document addressing each comment point by point, with responses highlighted in red;

• A marked-up version of the manuscript highlighting all changes;

• A clean version of the revised manuscript.

We thank you and the reviewers for your constructive feedback and consideration of our work. We hope that the revised version meets the journal’s expectations and we remain at your disposal for any further clarification.

Kind regards,

David Zarka, PhD

on behalf of all authors

Attachment Submitted filename: PLOS_Response_to_Reviewers.docx

# Editor report: Decision Letter 1

**DOI:** 10.1371/journal.pone.0359658.r003

**Authors:** Jenny Wilkinson

16 Sep 2026

Twenty years of university-graduated osteopaths in Belgium: a cross-sectional survey

PONE-D-26-07875R1

Dear Dr. Zarka,

We’re pleased to inform you that your manuscript has been judged scientifically suitable for publication and will be formally accepted for publication once it meets all outstanding technical requirements.

Within one week, you’ll receive an e-mail detailing the required amendments. When these have been addressed, you’ll receive a formal acceptance letter and your manuscript will be scheduled for publication.

An invoice will be generated when your article is formally accepted. Please note, if your institution has a publishing partnership with PLOS and your article meets the relevant criteria, all or part of your publication costs will be covered. Please make sure your user information is up-to-date by logging into Editorial Manager at Editorial Manager® and clicking the ‘Update My Information' link at the top of the page. For questions related to billing, please contact billing support.

If your institution or institutions have a press office, please notify them about your upcoming paper to help maximize its impact. If they’ll be preparing press materials, please inform our press team as soon as possible -- no later than 48 hours after receiving the formal acceptance. Your manuscript will remain under strict press embargo until 2 pm Eastern Time on the date of publication. For more information, please contact onepress@plos.org.

Kind regards,

Jenny Wilkinson, PhD

Academic Editor

PLOS One

Additional Editor Comments (optional):

Thank you for providing revisions and your patience while we waited on responses from reviewers. The responses and revised manuscript have satisfactorily address reviewer comments.

Reviewers' comments:

# Editor report: Acceptance letter

**DOI:** 10.1371/journal.pone.0359658.r004

**Authors:** Jenny Wilkinson

PONE-D-26-07875R1

PLOS One

Dear Dr. Zarka,

I'm pleased to inform you that your manuscript has been deemed suitable for publication in PLOS One. Congratulations! Your manuscript is now being handed over to our production team.

At this stage, our production department will prepare your paper for publication. This includes ensuring the following:

* All references, tables, and figures are properly cited

* All relevant supporting information is included in the manuscript submission,

* There are no issues that prevent the paper from being properly typeset

You will receive further instructions from the production team, including instructions on how to review your proof when it is ready. Please keep in mind that we are working through a large volume of accepted articles, so please give us a few days to review your paper and let you know the next and final steps.

Lastly, if your institution or institutions have a press office, please let them know about your upcoming paper now to help maximize its impact. If they'll be preparing press materials, please inform our press team within the next 48 hours. Your manuscript will remain under strict press embargo until 2 pm Eastern Time on the date of publication. For more information, please contact onepress@plos.org.

You will receive an invoice from PLOS for your publication fee after your manuscript has reached the completed accept phase. If you receive an email requesting payment before acceptance or for any other service, this may be a phishing scheme. Learn how to identify phishing emails and protect your accounts at https://explore.plos.org/phishing.

If we can help with anything else, please email us at customercare@plos.org.

Thank you for submitting your work to PLOS One and supporting open access.

Kind regards,

PLOS One Editorial Office Staff

on behalf of

Dr Jenny Wilkinson

Academic Editor

PLOS One
